# Poly(I:C) Induces Human Lung Endothelial Barrier Dysfunction by Disrupting Tight Junction Expression of Claudin-5

**DOI:** 10.1371/journal.pone.0160875

**Published:** 2016-08-09

**Authors:** Li-Yun Huang, Christine Stuart, Kazuyo Takeda, Felice D’Agnillo, Basil Golding

**Affiliations:** 1 Laboratory of Plasma Derivatives, Division of Hematology Research and Review, Center for Biologics Evaluation and Research, Food and Drug Administration, Silver Spring, Maryland, United States of America; 2 Laboratory of Biochemistry and Vascular Biology, Division of Hematology Research and Review, Center for Biologics Evaluation and Research, Food and Drug Administration, Silver Spring, Maryland, United States of America; 3 Microscopy and Imaging Core Facility, Center for Biologics Evaluation and Research, Food and Drug Administration, Silver Spring, Maryland, United States of America; Hungarian Academy of Sciences, HUNGARY

## Abstract

Viral infections are often accompanied by pulmonary microvascular leakage and vascular endothelial dysfunction via mechanisms that are not completely defined. Here, we investigated the effect of the Toll-like receptor 3 (TLR3) ligand polyinosinic-polycytidylic acid [Poly(I:C)], a synthetic analog of viral double-stranded RNA (dsRNA) commonly used to simulate viral infections, on the barrier function and tight junction integrity of primary human lung microvascular endothelial cells. Poly(I:C) stimulated IL-6, IL-8, TNFα, and IFNβ production in conjunction with the activation of NF-κB and IRF3 confirming the Poly(I:C)-responsiveness of these cells. Poly(I:C) increased endothelial monolayer permeability with a corresponding dose- and time-dependent decrease in the expression of claudin-5, a transmembrane tight junction protein and reduction of *CLDN5* mRNA levels. Immunofluorescence experiments revealed disappearance of membrane-associated claudin-5 and co-localization of cytoplasmic claudin-5 with lysosomal-associated membrane protein 1. Chloroquine and Bay11-7082, inhibitors of TLR3 and NF-κB signaling, respectively, protected against the loss of claudin-5. Together, these findings provide new insight on how dsRNA-activated signaling pathways may disrupt vascular endothelial function and contribute to vascular leakage pathologies.

## Introduction

Endothelial cells (EC) are critical in maintaining the integrity of the circulatory system [1, 2]. During infection, activated ECs release cytokines and undergo morphological changes. Changes in endothelial tight junctions (TJs) and adherens junctions (AJs) following infection and inflammation typically lead to increased vascular permeability [2]. The latter changes play an important role in the pathogenesis of septic shock, and possibly in acute lung injury [2]. TJs are composed of transmembrane proteins including occludin, claudins (primarily claudin-3 and -5), junctional adhesion molecules and cytoplasmic ZO-family members. VE-cadherin, the major component of AJs, plays a key role in regulating endothelial barrier function as evidenced by the increased permeability caused by the disruption of interendothelial AJs [3].

Viral diseases such as influenza and dengue fever are often accompanied by pulmonary microvascular leakage and endothelial dysfunction [4–7]. The host response to viral infection is partly regulated by receptors that recognize viral nucleic acids including double-stranded RNA (dsRNA) [8–11]. Exposure to dsRNA occurs extracellularly after cell lysis or intracellularly through infection with viruses that have dsRNA genomes or with dsDNA viruses that undergo a dsRNA stage during replication [8]. Several families of proteins recognize dsRNA, including membrane/endocytic Toll-like receptor 3 (TLR3), the dsRNA-dependent protein kinase (PKR), cytosolic RNA helicases such as retinoic acid inducible gene-I (RIG-I) and the melanoma differentiation-associated gene 5 (MDA5) [12]. Binding of dsRNA to TLR3 recruits the adaptor molecule TIR-domain-containing adapter-inducing interferon-β (TRIF), while binding to cytosolic RIG-I and MDA5 recruits the adapter molecule mitochondrial antiviral-signaling protein (MAVS). Both pathways activate the antiviral interferon (IFN) response via interferon regulatory factor 3 (IRF3) and NF-κB [12, 13]. Uncontrolled or sustained innate immune response via TLR3 has been shown to contribute to morbidity and mortality in certain viral infection models, suggesting that the modulation of the TLR3 pathway may offer an attractive strategy to protect against a variety of diseases [14–16].

Poly(I:C) is a synthetic dsRNA compound that has been shown to recapitulate many of the effects of viral dsRNA *in vitro* and *in vivo*, and thus has commonly been used to simulate viral infections [17, 18]. In this study, we examine the effect of Poly(I:C) on the permeability and inflammatory response of primary human lung endothelial cells. Our findings suggest a profound effect of Poly(I:C) on the downregulation of endothelial tight junction protein claudin-5 that correlates with a cell death-independent disruption in barrier function.

## Material and Methods

### Reagents

Poly(I:C) HMW (synthetic double-stranded RNA with high molecular weight) was purchased from InvivoGen (San Diego, CA). IκBα inhibitor Bay11-7082 was purchased from EMD Chemicals (Gibbstown, NJ). Endosomal acidification inhibitor chloroquine (CQ), cycloheximide, and albumin-fluorescein isothiocyanate conjugate (albumin-FITC) were purchased from Sigma Chemical Co. (St. Louis, MO). Recombinant human interferon beta 1a (catalog #11415–1) was purchased from PBL Assay Science (Piscataway, NJ). Recombinant human interleukin-6 (catalog #rhil-6) was obtained from InvivoGen.

### Antibodies

Rabbit polyclonal antibody against human claudin-5 (catalog #34–1600), mouse monoclonal antibody against human claudin-5 (catalog #35–2500), and mouse monoclonal antibody against human ZO-1 (catalog #33–9100) were purchased from Invitrogen (Life Technology Corp., Grand Island, NY). Rabbit monoclonal antibody against human LAMP-1 (catalog #9091), rabbit monoclonal antibody against NF-κB p65 (catalog #8242), rabbit monoclonal antibody against VE-cadherin (catalog #2500), rabbit polyclonal antibody against human IκBα (catalog #9242), rabbit polyclonal antibody against human RIG-I (catalog #4520), rabbit polyclonal antibody against human MDA5 (catalog #5321), and mouse monoclonal antibody against phospho-NF-κB p65 (Ser536) (catalog #3036) were purchased from Cell Signaling Technology (CST, Danvers, MA). Goat polyclonal anti-human TLR3 (catalog #AF1487) was purchased from R&D Systems (Minneapolis, MN). Rabbit polyclonal anti-human TRIF (catalog #ALX-215-016-R200) was purchased from Enzo Life Sciences, Inc. (Farmingdale, NY). Rabbit monoclonal antibodies against human phosphorylated-IRF3 (catalog #ab76493) and IRF3 (catalog #ab76409) were purchased from Abcam (Cambridge, MA). Goat polyclonal anti-human VE-cadherin (catalog #sc-6458), rabbit polyclonal anti-human β-tubulin antibody (catalog #sc-9104), and rabbit polyclonal anti-NF-κB p65 antibody (catalog #sc-7151) were purchased from Santa Cruz Biotechnology (Santa Cruz, CA). Donkey anti-rabbit IgG-horse radish peroxidase (HRP) (catalog #sc-2313), donkey anti-goat IgG-HRP (catalog #sc-2020) and goat anti-mouse IgG-HRP (catalog #sc-2005) were purchased from Santa Cruz Biotechnology. Goat anti-rabbit IgG-HRP (catalog #7074) and horse anti-mouse IgG-HRP (catalog #7076) were purchased from CST.

### Endothelial cell culture and treatment

Primary human lung microvascular endothelial cells (HLECs; catalog #CC-2815) were purchased from Lonza (Walkersville, MD). HLECs were cultured in EBM basal medium (catalog #CC-3129) with EGM-2MV supplements (catalog #CC-4147, Lonza) and Glutamax (Invitrogen). Cells were cultured at a final concentration of 1–2 x10^5^ cells/mL, grown to confluence, and then stimulated with Poly(I:C) at various concentrations for 3–24 h. For inhibitor experiments, cells were pre-treated with inhibitors for 1 h before the addition of Poly(I:C). Following stimulation, culture supernatants were collected at different incubation times for cytokine/chemokine ELISAs and whole cell lysates were collected for Western blot analysis. IL-6, IL-8, TNFα and IFNβ were measured using commercial ELISA kits from R&D Systems (Minneapolis, MN) and Pierce Biotechnology (Thermo Scientific, Rockford, IL). The concentration and absorbance values were expressed as the means of duplicate or triplicate samples with standard errors. All data shown are representative from three or more experiments.

### Western blot analysis

HLECs were lysed in ice-cold RIPA buffer (50 mM Tris, 150 mM NaCl, 1% IgePal-630, 0.5% deoxycholate, 1 mM EDTA) containing a protease inhibitor mixture (Cocktail Set III) and phosphatase inhibitors (Cocktail Set V) (EMD Millipore, Billerica, MA). Following centrifugation, whole cell supernatants were collected and stored at -80˚C. Protein concentration was measured using the BCA assay (Pierce). Equal protein amounts (4–6 μg) from the stimulated HLEC lysates were separated by sodium dodecyl sulfate-polyacrylamide gel electrophoresis (SDS-PAGE) using NuPAGE 4–12% Bis-Tris or 3–8% Tris-Acetate mini-gels (Invitrogen) under denaturing conditions. The separated proteins were electroblotted onto polyvinylidene difluoride membranes (pore size, 0.45 μm; Invitrogen). Protein blots were blocked for 1 h in TBS containing 0.1% Tween 20 (TBST) with 5% nonfat dry milk or 5% BSA and probed with the specific primary antibody followed by HRP-conjugated secondary antibody. Immunodetection was revealed on HyperECL film with the ECL Plus chemiluminescence detection system (GE Healthcare UK Ltd., Buckinghamshire, United Kingdom) according to the manufacturer's instructions. The relative amount of protein was estimated by densitometry analysis with Image J software (National Institutes of Health, Bethesda, MD).

### Transendothelial electrical resistance (TEER) measurement

HLECs were grown to confluence on porous polyester membrane inserts (12 mm diameter, 0.4 μm pore size; Transwell, Corning, Cambridge, MA). The upper and lower compartments contained 0.5 and 1.5 mL of media, respectively. Monolayers were either left untreated or Poly(I:C) was added to the upper compartment at 0.1, 1 or 10 μg/mL. TEER measurements were performed using an EVOM volt-ohmmeter connected to a 12-mm Endohm unit (World Precision Instruments, Sarasota, FL). A decrease in TEER indicates an increase in monolayer permeability, whereas an increase in TEER signifies an increase in monolayer integrity. Data were collected from triplicate inserts per treatment in each experiment. Values were reported as the percentage of basal TEER obtained by dividing the resistance values of each treated monolayer by the resistance value of the control monolayer at each given treatment.

### Albumin permeability assay

HLECs cultured to confluence on porous membrane inserts in Transwell chambers were treated with various concentrations of Poly(I:C). After 24 h, 50 μL of culture medium from the upper chamber was replaced with an equal amount of medium containing 5 mg/mL albumin-FITC (final concentration of 500 μg/mL). After 2 h, permeability across the cell monolayer was analyzed by determining the amount of albumin-FITC in the lower compartment. 20 μL samples were drawn from the lower chamber and diluted 10-fold. Fluorescence measurements were obtained using a microplate reader (TECAN GENios; Research Triangle Park, NC) with excitation and emission filters of 485 and 535 nm, respectively. The amount of albumin-FITC leakage across the monolayer was calculated using an albumin-FITC standard curve. Data were collected from triplicate inserts per treatment in each experiment.

### Analysis of cell monolayer integrity and viability

Monolayer cell density was assessed by counting the number of adherent cells with normal nuclear morphology identified by dual staining with the membrane-permeable DNA fluorochrome Hoechst 33342 and propidium iodide (PI) (Invitrogen). This staining method distinguishes between normal, apoptotic, or necrotic cells. Normal cells were defined as those with no PI staining and without evidence of nuclear condensation. Apoptotic cells were defined as those with condensed or fragmented nuclei without PI staining. Necrotic cells were defined as those visibly stained by PI indicative of cell membrane lysis. Briefly, HLECs were grown to confluence in 24-well plates to form adherent monolayers and treated with Poly(I:C) at 0.1, 1 and 10 μg/mL for 24 h. The adherent monolayers were then incubated with 1 μg/mL Hoechst 33342 and 1 μg/mL PI for 20 min at 37^◦^C. Photomicrographs of five separate fields per well (top, bottom, right, left, and center) were obtained using an Olympus IX71 inverted microscope with a 20x objective lens (Olympus America, Melville, NY) and Olympus DP80 digital camera system. Digital images were analyzed using Image J. For each treatment, cell monolayer density was calculated as the percentage of adherent cells with normal nuclear morphology relative to untreated cultures performed in parallel. To further assess the viability of Hoechst-labeled adherent cells, cultures were washed in HBSS containing 0.1% bovine serum albumin, then incubated with Hoechst 33342 and 2 μM Calcein-AM (Life Technologies) for 10 min at room temperature. Following incubation, the monolayers were washed once and photomicrographs were obtained as described above. Calcein AM is a membrane-permeable dye that following conversion by intracellular esterases is retained inside live cells as a membrane-impermeable Calcein fluorescent dye.

### Real-time PCR

RNA from HLECs was collected using the RNeasy Mini kit (Qiagen, Hilden, Germany) and converted to cDNA using the TaqMan reverse transcription kit (Applied Biosystems, Waltham, MA) according to the manufacturer’s protocol. Gene expression was analyzed using TaqMan Fast Universal 2x PCR Master Mix (No AmpErase UNG) and TaqMan gene expression assays for *GAPDH* (Hs99999905_m1), *CLDN5* (Hs01561351_m1), *TJP1* (Hs01551861_m1), and *CDH5* (Hs00901469_m1) (Applied Biosystems). Reactions were performed in triplicate and run on the Applied Biosystems ViiA^TM^ 7 real-time PCR system. Fold gene expression relative to *GAPDH* as a reference gene was calculated using the 2 ^- ΔΔCT^ method [19].

### Immunolabeling and confocal microscopy

HLECs were grown to confluence in Nunc Lab-Tek chambered coverglass or slides (Thermo Scientific, Waltham, MA). Cells were treated with 1 or 10 μg/mL Poly(I:C) for 24 h. Cells were then fixed with 4% paraformaldehyde or ice cold methanol for 10 min and incubated with 5% normal donkey serum for 30 min at room temperature. Cells were subsequently incubated with primary antibodies against LAMP-1 and claudin-5 (catalog #35–2300, Invitrogen), VE-cadherin (Santa Cruz Biotechnology), ZO-1, or NF-κB p65 (Santa Cruz Biotechnology) for 2 h, followed by incubation with secondary antibodies conjugated with Alexa Fluor® 488 with or without Alexa Fluor® 594 (Invitrogen). AF488 Phalloidin (Thermo Scientific) was used to stain F-actin and nuclei were stained using Hoechst 33342. Fluorescence microscopic images were obtained by Leica TCS_SP8 DMI6000 confocal microscope system (Leica Microsystems, Mannheim, Germany). Images were acquired using a 63x objective lens (N.A. 1.4) for Alexa 488 and 594 emission wavelengths and stored in lif format for further analysis. Fluorescence intensity and co-localization analyses were performed using the Leica LASAF and Bitplane Imaris software. Briefly, mean fluorescence intensity was measured by averaging readings from 15 randomly selected images per treatment for a minimum of three separate experiments. Pearson’s co-localization coefficient was calculated from images collected from a minimum of three separate experiments.

For analysis of NF-κB translocation, fluorescence images were obtained using an Olympus IX71 inverted microscope with a 40x objective lens and Olympus DP80 digital camera system (Olympus America, Melville, NY).

### Statistical analyses

The results are presented as means ± SE. The data were analyzed by the two-tailed unpaired *t*-test at the 95% confidence interval using Prism (version 6) software for Windows (GraphPad Software, San Diego, CA). *p*<0.05 was considered statistically significant.

## Results

### Poly(I:C) promotes cytokine and chemokine release from HLEC

**Fig 1:**
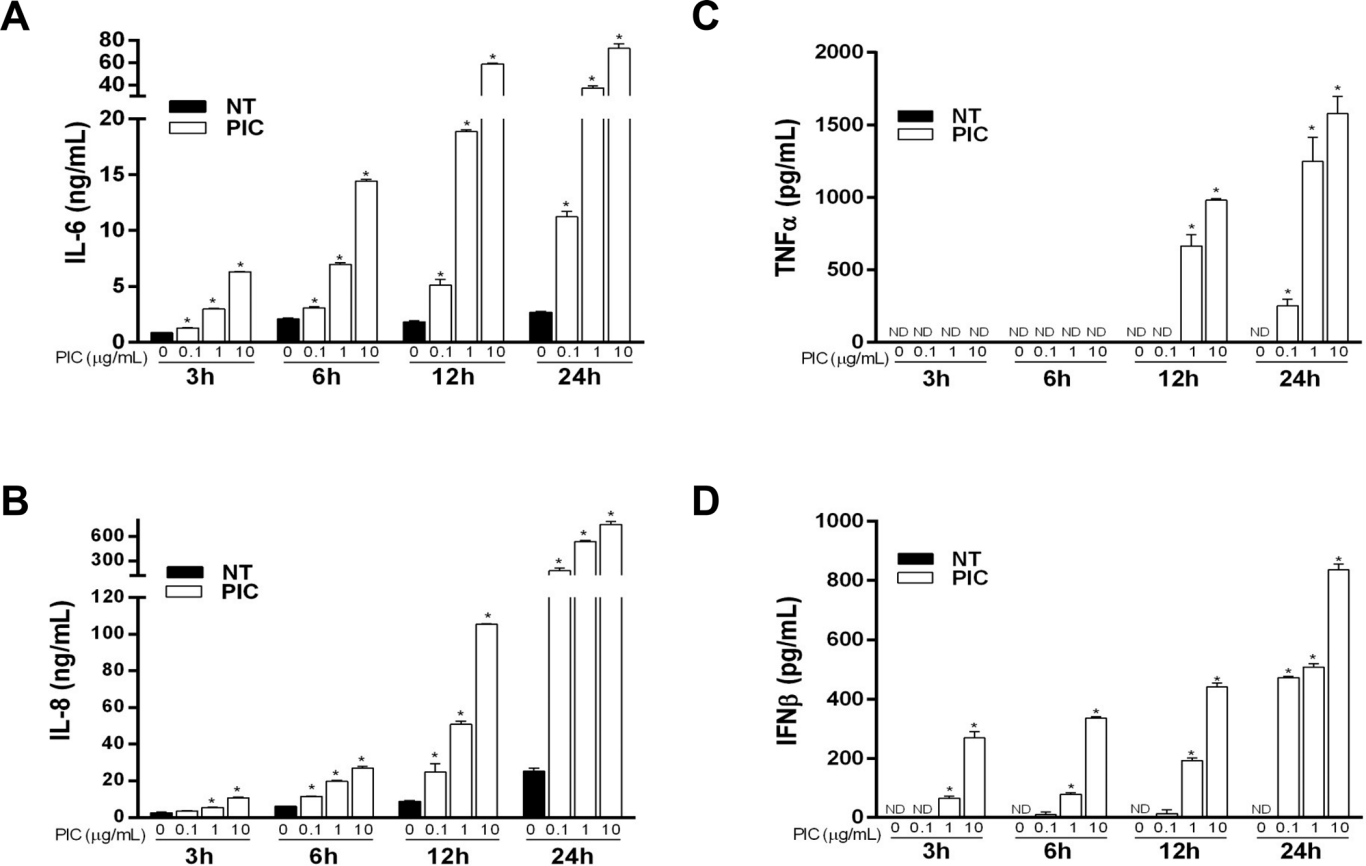
Poly(I:C) induces cytokine and chemokine production in HLECs. Cells were treated with medium (NT) or Poly(I:C) (PIC) at 0.1, 1 and 10 μg/mL as indicated. Supernatants were harvested 3, 6, 12, and 24 h after treatment and assayed for IL-6 (A), IL-8 (B), TNFα (C), and IFNβ (D) secretion by ELISA. Data shown as means ± SE are representative of those from at least three similar experiments. *, *p*<0.05 versus control within each time point. ND = not detected.

TLR3 signaling activates transcription factors IRF3 and NF-κB that upregulate the synthesis of multiple cytokines and chemokines in a number of different cell types [12]. To confirm that HLECs are responsive to TLR3 agonists, we measured the dose- and time-dependent production of cytokines and chemokines following treatment with Poly(I:C) (Fig 1). Dose-dependent increases in IL-6, IL-8, and IFNβ were detected as early as 3 h, whereas increased TNFα release occurred at later time points (i.e., 12 and 24 h) (Fig 1A–1D). These results confirm that the HLECs used in this study respond to the TLR3 agonist, Poly(I:C).

### Poly(I:C) increases HLEC monolayer permeability

**Fig 2:**
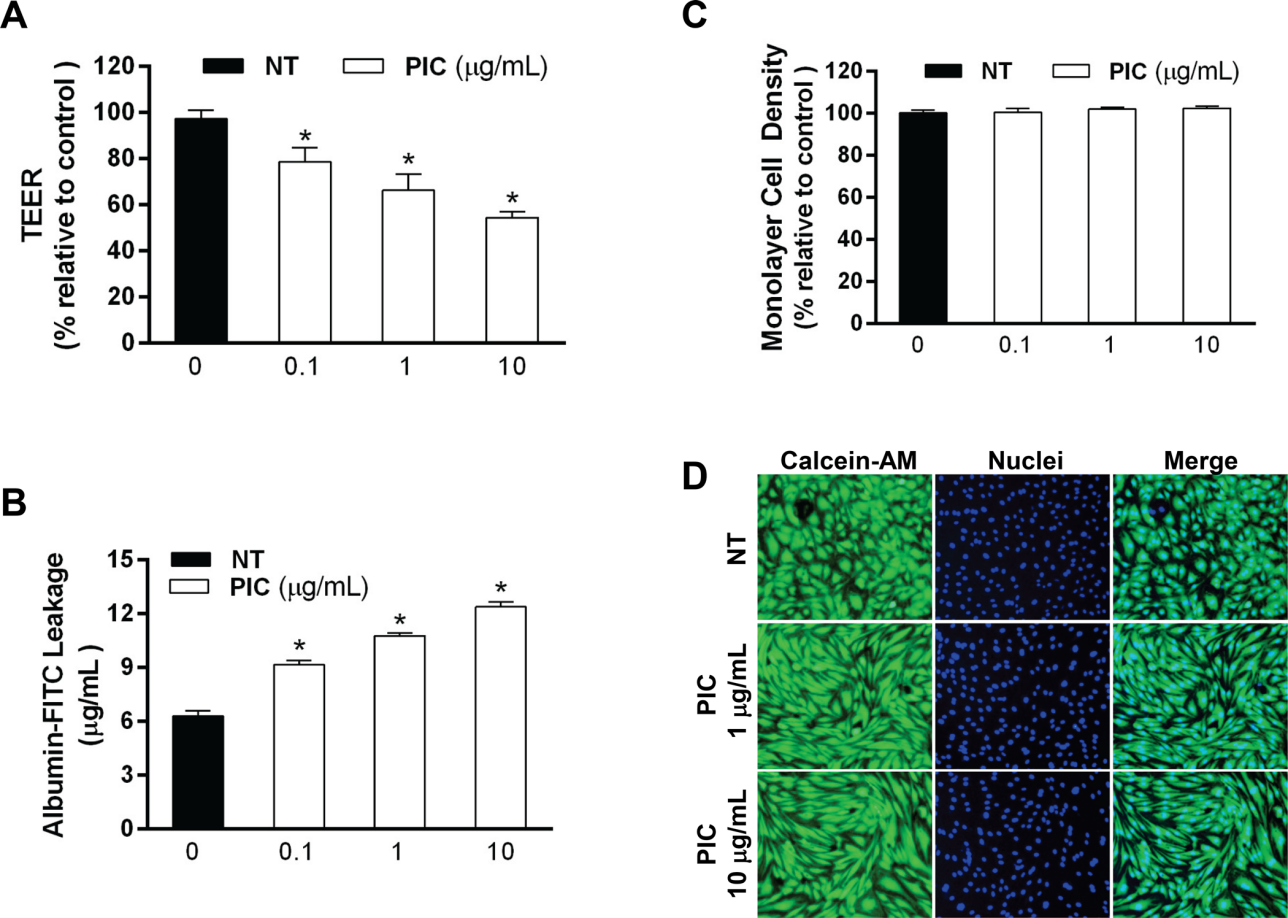
Poly(I:C) induces loss of barrier function. Confluent HLECs on porous membrane inserts in Transwell chambers were treated with Poly(I:C) at 0.1, 1, or 10 μg/mL for 24 h. Barrier function was assessed by measuring TEER (A) and monolayer permeability to albumin-FITC (B). (A) TEER values are reported as the percentage of basal TEER obtained by dividing the resistance values of each treated monolayer by the resistance value of the control monolayer. Means ± SE for three separate experiments are shown. *, *p*<0.05 versus control. (B) Albumin-FITC leakage across the monolayer was calculated using an albumin-FITC standard curve. Means ± SE for three separate experiments are shown. *, *p*<0.05 versus control. (C) Poly(I:C) does not alter monolayer cell density after 24 h stimulation. Monolayers were treated with medium alone or Poly(I:C) for 24 h, then stained with Hoechst 33342 and PI. For each treatment, immunofluorescence images of five separate fields were visualized. For each image, adherent cells with normal nuclei were counted and the average was tabulated for each well. Monolayer cell density was calculated as the percentage of normal adherent nuclei relative to untreated control wells. Data from triplicate experiments are shown as means ± SE. (D) Calcein-AM viability staining. Monolayers were treated with medium alone or Poly(I:C) at 1 and 10 μg/mL for 24 h, then stained with Calcein-AM (green) and Hoechst 33342 as described in the Materials and Methods.

The effect of Poly(I:C) on EC permeability was measured by analyzing transmembrane endothelial electrical resistance (TEER) and the passage of albumin-FITC across the monolayer. Poly(I:C) induced a dose-dependent decrease in TEER and increase in the passage of albumin across HLEC monolayers indicating decreased endothelial barrier function (Fig 2A and 2B). Poly(I:C) 10 μg/mL reduced TEER as early as 6 h (53.2 ± 3.2%) and 12 h (41.6 ± 3.6%) relative to untreated controls. To examine whether changes in monolayer density or cell death could account for the observed barrier dysfunction, cultures were stained with Hoechst 33342 and PI for the identification and enumeration of adherent cells with normal nuclear morphology while excluding apoptotic and necrotic cells. After 24 h, Poly(I:C) did not cause any significant change in monolayer density compared to untreated cultures at any of the indicated doses (Fig 2C). In addition, PI-positive cells were not observed in the adherent monolayer. Calcein-AM staining further confirmed the viability of the Hoechst 33342-labeled adherent cells (Fig 2D). These data suggest that the Poly(I:C)-mediated increase in monolayer permeability is not the result of cell death or monolayer cell loss but rather an effect on the integrity of endothelial junctions.

### Poly(I:C) down-regulates claudin-5 expression in endothelial tight junctions

**Fig 3:**
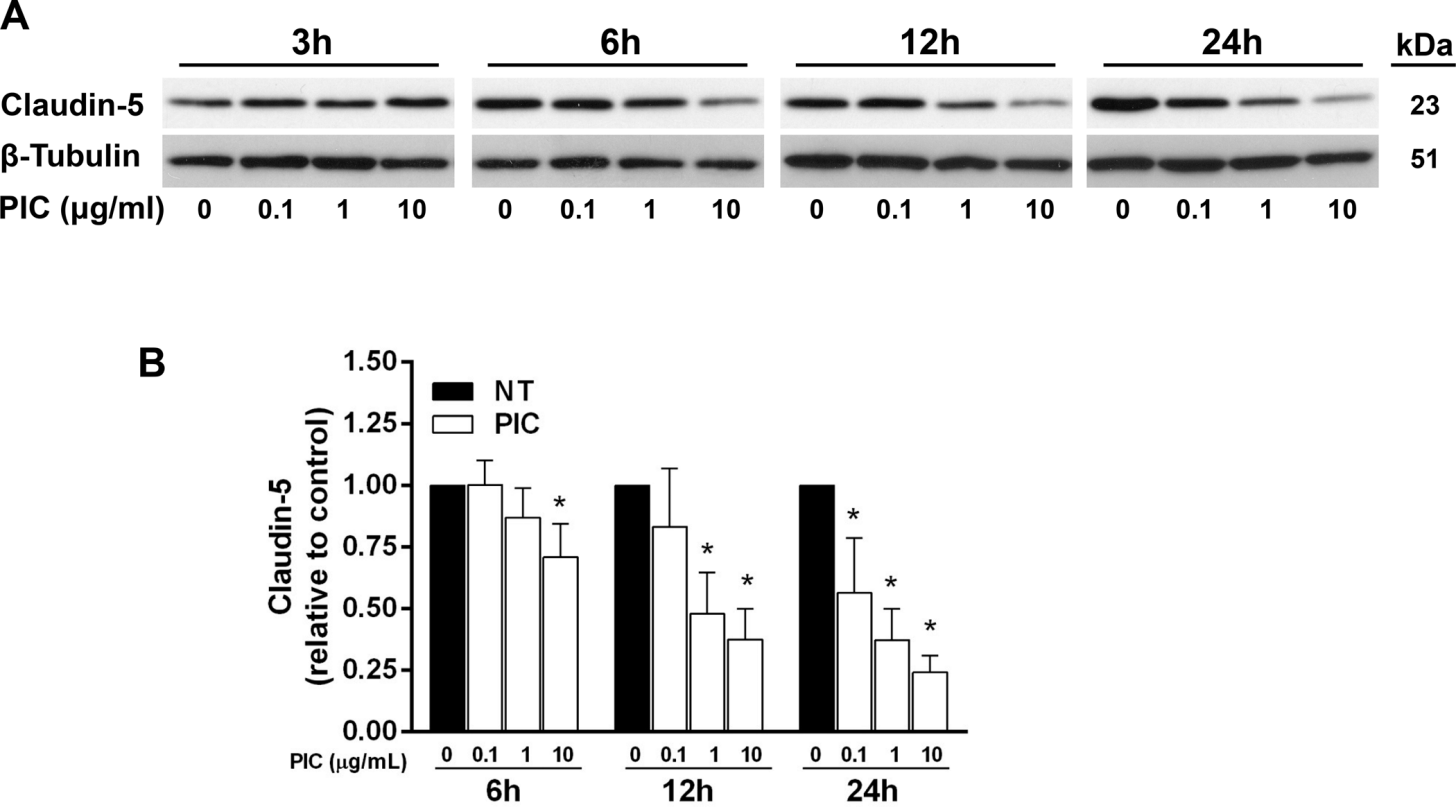
Poly(I:C) reduces claudin-5 expression in a time- and dose-dependent manner. HLEC were treated with medium alone or various doses of Poly(I:C). (A) Whole cell lysates collected at 3, 6, 12, and 24 h were analyzed for claudin-5 expression by Western Blot. β-tubulin served as a loading control. The immunoblot shown is representative of three separate experiments. (B) Claudin-5 expression was normalized to β-tubulin and is presented relative to control at each time point. Data shown are the means ± SE from at least four blots performed for each dose at each time point. *, *p*<0.05 versus control.

**Fig 4:**
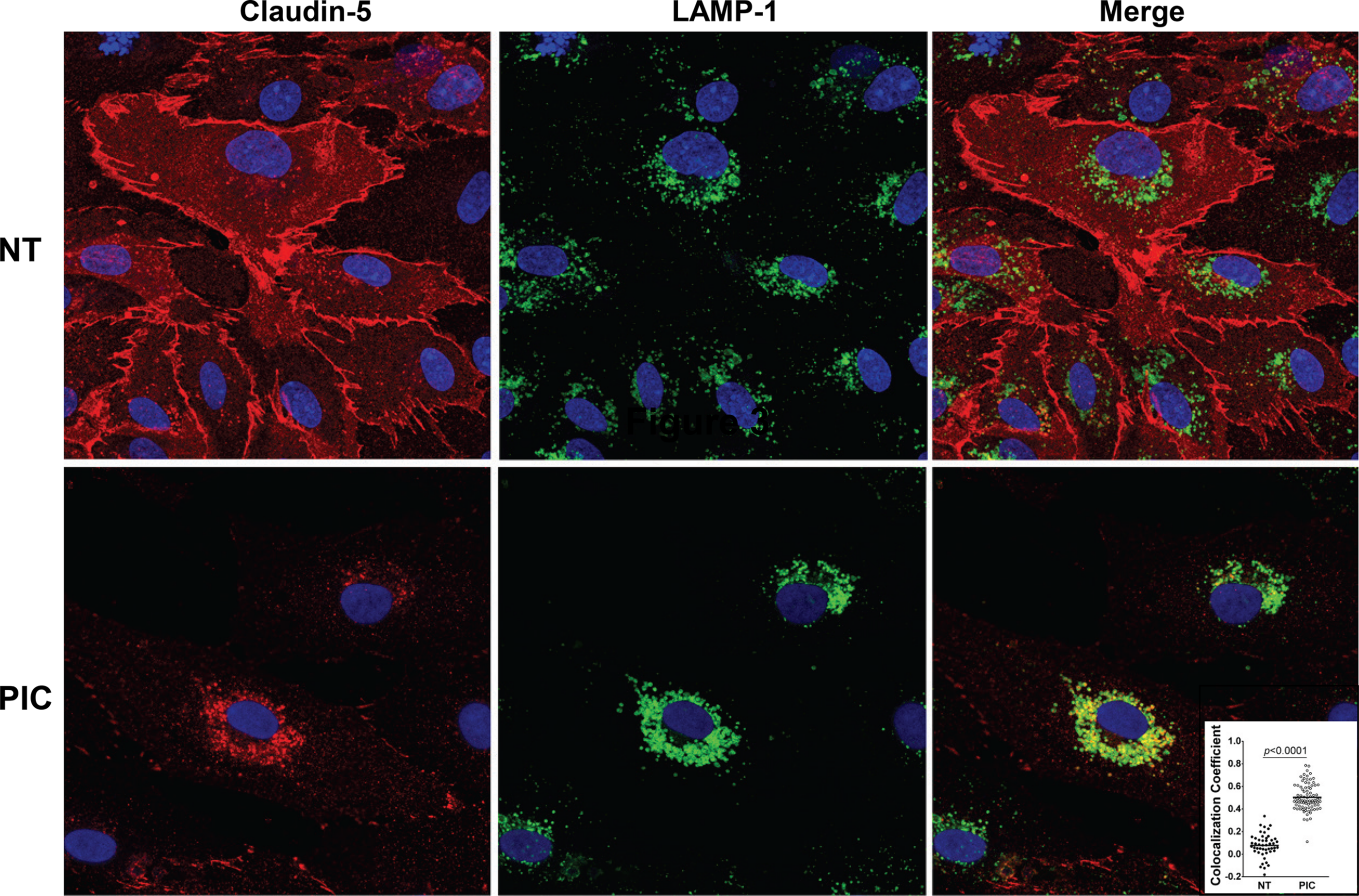
Poly(I:C) causes loss of interendothelial localization of claudin-5. HLECs grown on chamber slides were stimulated with medium (NT) or 1 μg/mL Poly(I:C) for 24 h. Immunofluorescence staining using antibodies against claudin-5 (red) and lysosomal marker LAMP-1 (green) showed claudin-5 expression at the plasma membrane and in lysosomes. Images are representative of three separate experiments (630x total magnification). *Inset*, the colocalization coefficient representing the degree of overlap between claudin-5 and LAMP-1 staining was calculated for images collected from a minimum of three separate experiments. Each data point in the graph represents one analyzed image.

The effect of Poly(I:C) on the expression of claudin-5, a key endothelial tight junction protein, was assessed by immunoblot and immunofluorescence. Immunoblot analyses showed that Poly(I:C) induced a dose- and time-dependent decrease in claudin-5 expression (Fig 3). Immunofluorescence analyses further confirmed the loss of claudin-5 immunoreactivity along interendothelial plasma membrane regions of HLECs (Fig 4). This loss of membrane-localized claudin-5 was accompanied by increased claudin-5 immunoreactivity in the peri-nuclear late endosomes/lysosomes as determined by co-localization with LAMP-1 (Fig 4). Poly(I:C) also induced progressive cellular elongation that correlated with the gradual appearance of central actin stress fibers that were absent at 6 h, detectable in a few monolayer regions at 12 h, and clearly widespread by 24 h at the 10 μg/mL concentration (S2 Fig). Additional experiments were performed to examine the effect of Poly(I:C) on the transmembrane adherens junction protein VE-cadherin and the linker protein ZO-1. Total expression levels of VE-cadherin were reduced at 24 h, but not at 6 or 12 h, while ZO-1 levels were unchanged (S1 Fig). Interendothelial staining for VE-cadherin and ZO-1 was reduced but to a lesser extent than claudin-5 at 24 h with 10 μg/mL Poly(I:C) (S2 Fig). Together, these findings suggest that the loss of claudin-5 likely plays an important role during the onset and progressive stages of Poly(I:C)-mediated barrier dysfunction, while changes in VE-cadherin may contribute to barrier dysfunction at the late time intervals.

### Poly(I:C) causes down-regulation of *CLDN5* mRNA

**Fig 5:**
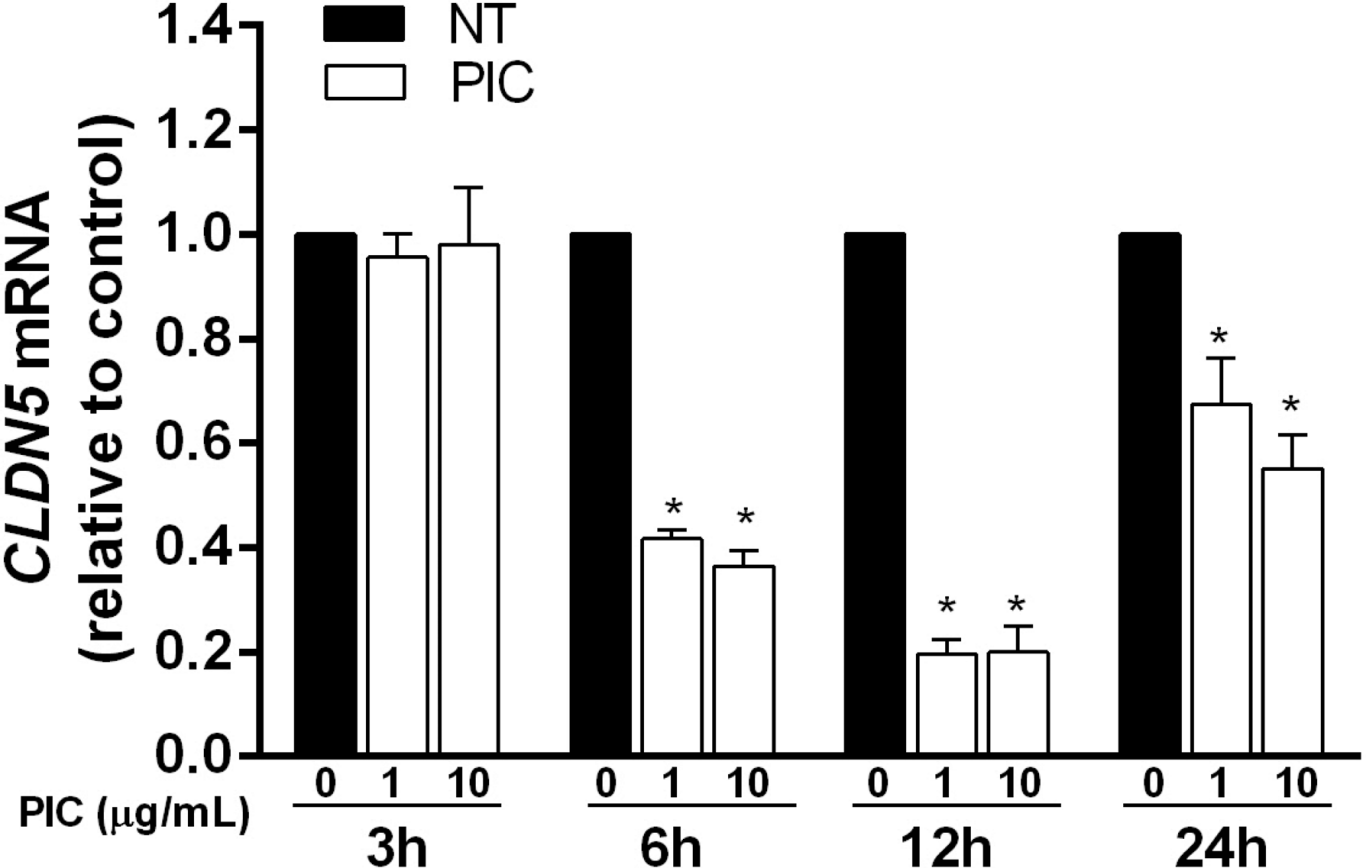
Poly(I:C) reduces *CLDN*5 mRNA in HLECs. Cells were treated with medium alone or Poly(I:C) at 1 and 10 μg/mL for 3, 6, 12 and 24 h. RNA was collected at each time point and analyzed for *CLDN5* gene transcript relative to *GAPDH* by real-time PCR. Data were collected from triplicate wells from three separate experiments for each dose at each time point and presented as means ± SE. *, *p*<0.05 versus control.

To investigate the pathway involved in Poly(I:C)-induced down-regulation of claudin-5, total cellular *CLDN5* mRNA levels were assessed. Poly(I:C) at 1 and 10 μg/mL reduced *CLDN5* mRNA at 6, 12, and 24 h with a slight recovery noted at the latter time point (Fig 5). In contrast, Poly(I:C) did not reduce VE-cadherin and ZO-1 transcript levels providing further evidence that the effects of Poly(I:C) on claudin-5 may be distinct from those on VE-cadherin and ZO-1 (S1 Fig). Additional cycloheximide chase experiments showed that the extent of Poly(I:C)-induced claudin-5 degradation compared to control in the presence or absence of cycloheximide was similar suggesting that Poly(I:C)-mediated loss of claudin-5 is not driven by enhanced degradation of claudin-5 (S3 Fig). Together, these findings suggest that the effects of Poly(I:C) on *CLDN5* gene transcription may play a primary role in the reduction of claudin-5 protein levels.

### Poly (I:C) signaling through TRIF, NF-κB, and IRF3

**Fig 6:**
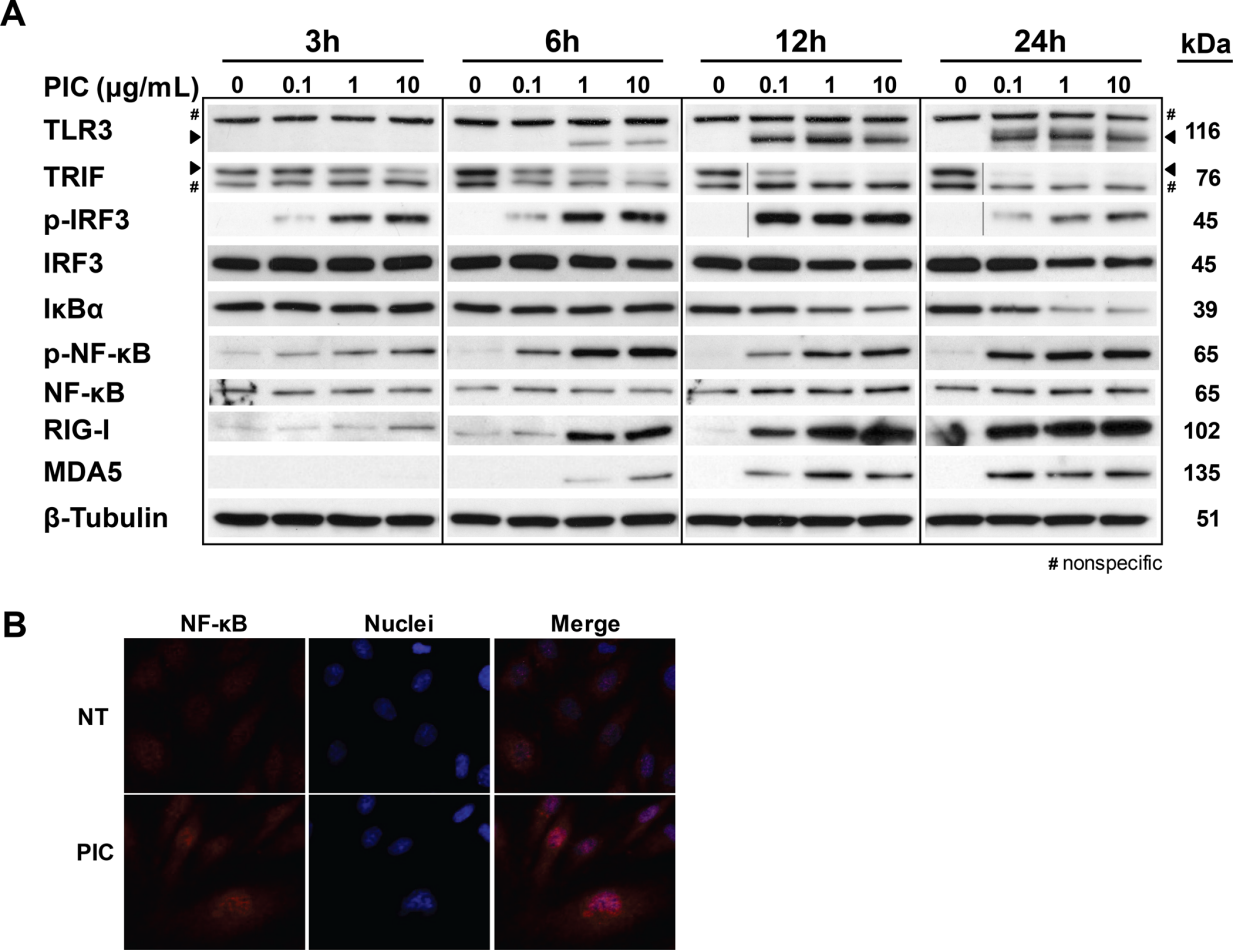
Poly(I:C) activates the TLR3/TRIF signaling. (A) HLECs were stimulated with Poly(I:C) at 0.1, 1 and 10 μg/mL for 3, 6, 12 and 24 h. Whole cell lysates were analyzed for TLR3, TRIF, phosphorylated-IRF3, total IRF3, IκBα, phosphorylated-NF-κB, NF-κB, RIG-I, MDA5, and β-tubulin (as a loading control) by Western blot. Arrowheads indicate TLR3 (116 kDa, lower band) and TRIF (76 kDa, top band). #, indicates a nonspecific band. Immunoblots are representative of three separate experiments that all showed similar results. (B) Poly(I:C) induces nuclear translocation of NF-κB. HLEC were stimulated with medium (NT) or 5 μg/mL Poly(I:C) for 24 h. Immunofluorescence staining was performed using antibodies against NF-κB (red) and nuclei were counterstained with Hoechst 33342 (blue). Images are representative of three separate experiments (400x total magnification).

To assess the potential relationship between claudin-5 down-regulation and the TLR3 pathway, we examined the effect of Poly(I:C) on the downstream targets of TLR3 signaling. Poly(I:C) increased the expression of full-length TLR3 (116 kDa band) and produced a disappearance of TRIF (76 kDa band) in a dose- and time-dependent manner (Fig 6A). The Poly(I:C)-mediated loss of TRIF has been previously reported but it is not clear if this reflects degradation or an induced protein modification that the antibody is unable to recognize [20]. Similar to TLR3 induction, Poly(I:C) increased the total expression levels of RIG-I and MDA5. Consistent with the activation of TLR3-TRIF signaling, Poly(I:C) increased the phosphorylation of IRF3 and NF-κB, and produced a gradual reduction of total IκBα levels. Nuclear translocation of NF-κB was confirmed by immunofluorescence analyses (Fig 6B).

**Fig 7:**
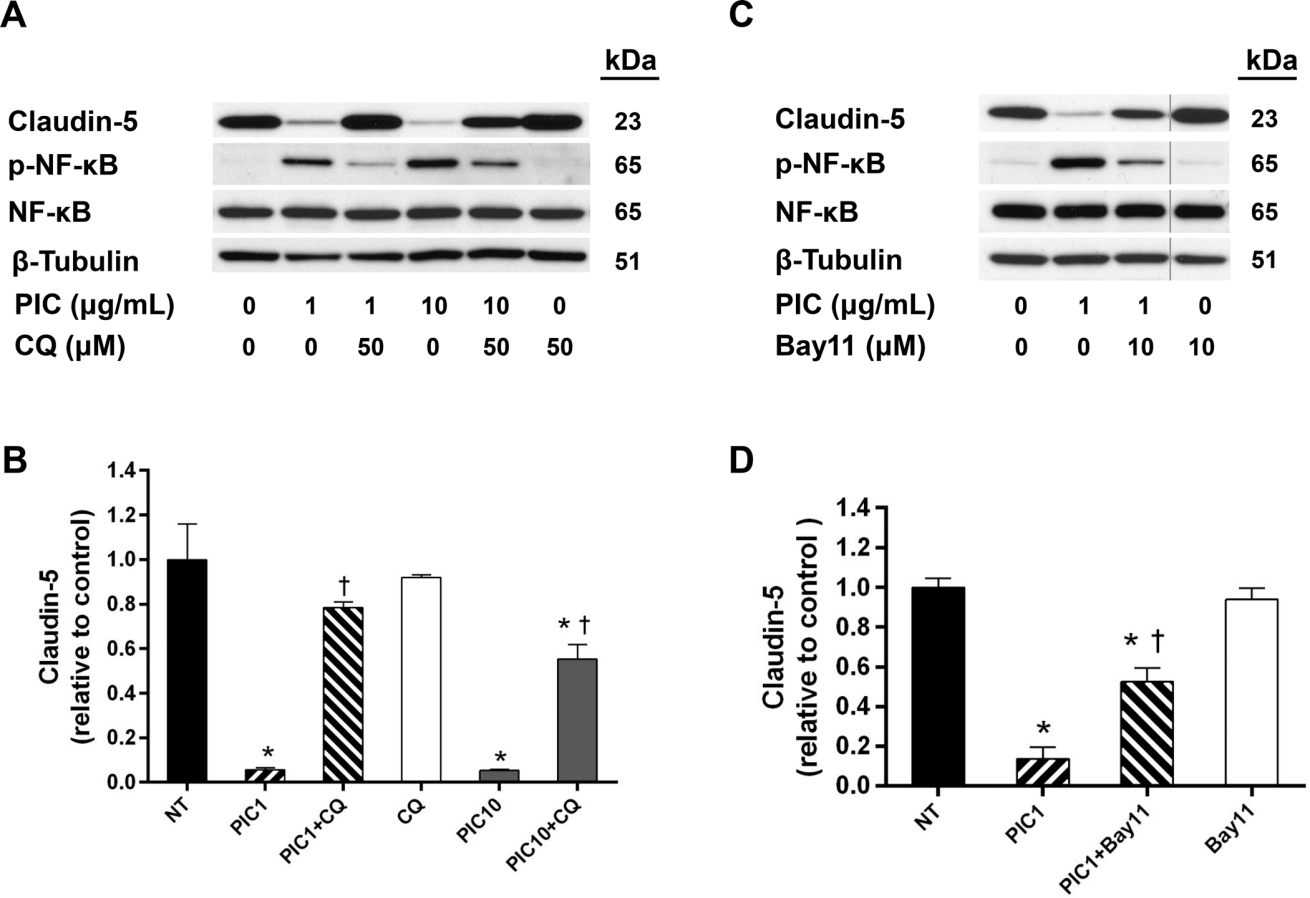
Poly(I:C)-induced down-regulation of claudin-5 inhibited by CQ and Bay 11–7082. HLECs were pre-treated with inhibitors for 1 h prior to 24 h incubation with Poly(I:C). Whole cell lysates were collected from cells treated with Poly(I:C) in the presence or absence of (A) 50 μM CQ (an endosomal acidification inhibitor) or (C) 10 μM Bay 11–7082 (Bay 11; an IκBα inhibitor) and analyzed for claudin-5, phosphorylated NF-κB, and NF-κB by Western blot. Blots are representative of three similar experiments. (B, D) Claudin-5 expression was normalized to tubulin and presented relative to control. Means ± SE for three separate experiments are shown. *, *p*<0.05 versus control; †, p<0.05 versus Poly(I:C) alone.

Poly(I:C) activation of TLR3-TRIF signaling requires acidification of the endosomal compartment [21]. Pretreatment with chloroquine (CQ), an inhibitor of endosomal/lysosomal acidification, prevented the Poly(I:C)-induced loss of claudin-5, suggesting a potential contributing role for TLR3-TRIF signaling (Fig 7A and 7B). Interestingly, CQ also inhibited Poly(I:C)-induced NF-κB phosphorylation suggesting a possible link between NF-κB and claudin-5 down-regulation as reported by others [22–24]. As further support for the involvement of NF-κB, pretreatment with Bay11-7082, an inhibitor of NF-κB signaling, attenuated the Poly(I:C)-induced reduction of claudin-5 (Fig 7C and 7D).

## Discussion

In this study, Poly(I:C) was used as a surrogate for viral dsRNA expressed during infection (e.g. dengue and influenza) to characterize its effect on endothelial activation and integrity. Consistent with previous studies on other cell types, Poly(I:C) stimulated HLEC to secrete multiple cytokines and chemokines [12, 20, 25, 26]. Poly(I:C) induced a cell death-independent loss of barrier function that correlated with the disruption of endothelial TJs as evidenced by the reduced total expression levels and interendothelial localization of claudin-5. The loss of claudin-5 was accompanied by a significant down-regulation of *CLDN5* mRNA levels suggesting the downstream signaling pathways triggered by Poly(I:C) contributed to the transcriptional dysregulation of the claudin-5 gene.

Activation of TLR3-TRIF signaling requires endosomal acidification [21]. We found that pretreatment with CQ, an inhibitor of endosomal/lysosomal acidification, protected against Poly(I:C)-mediated claudin-5 down-regulation indirectly implicating TLR3-TRIF signaling in this response. TLR3-TRIF activated its downstream targets IRF3 and NF-κB as evidenced by the phosphorylation of IRF3 and NF-κB, down-regulation of IκBα, and nuclear translocation of NF-κB. Consistent with a potential role for NF-κB in the down-regulation of claudin-5, we found that pretreatment with Bay11-7082 protected against the loss of claudin-5. Other studies have also implicated NF-κB signaling in the downregulation of TJ molecules [22–24]. The present study focused on the TLR3-TRIF pathway; however, it is important to recognize that IRF3 and NF-κB are also downstream targets of the cytoplasmic dsRNA receptors, RIG-I and MDA5. We found that Poly(I:C) increased the total expression of RIG-I and MDA5, consistent with a previous study that implicated Poly(I:C)-mediated TLR3-TRIF signaling in this induction process [27]. However, the potential involvement of RIG-I and/or MDA5 in the loss of claudin-5 is not yet known and will be the subject of future studies.

Claudin-5 undergoes continuous internalization and recycling back to the plasma membrane through endosomal/lysosomal pathways [28]. Poly(I:C)-treated HLECs showed significant reduction of membrane-expressed claudin-5 while the remaining cytoplasmic-expressed claudin-5 localized primarily to late endosomes/lysosomes. We initially interpreted this finding as being the result of a Poly(I:C)-mediated enhancement of lysosomal claudin-5 degradation; however, our preliminary cycloheximide chase experiments suggest that Poly(I:C) does not increase the rate of claudin-5 disappearance. While these initial data suggest that Poly(I:C) does not enhance claudin-5 degradation, further studies will be required to examine whether Poly(I:C) treatment alters the trafficking of claudin-5 to and from the plasma membrane.

Previous studies have shown that cytokines/chemokines can alter the expression and/or localization of tight junction molecules [22–24]. In the present study, we show that Poly(I:C) induces several cytokine/chemokines including IL-6, TNFα, IFNβ, and IL-8. Our preliminary studies have shown HLECs treated with IL-6 or IFNβ, at concentrations similar to those secreted with 10 μg/mL Poly IC at late time intervals, failed to reduce total claudin-5 expression at 24 h (S4 Fig). The potential contribution of TNFα and IL-8 to the down-regulation of claudin-5 in this system also seems unlikely given their temporal release kinetics in relation to the early onset of Poly(I:C)-induced claudin-5 reduction (~6 h). Nevertheless, our current studies do not completely rule out the possibility that individual or combined autocrine effects of released cytokines/chemokines may have contributed to the downstream loss of claudin-5. Thus, further studies will be required to investigate their potential contribution.

We postulate that the pathogenesis of certain respiratory viral infections including avian influenza outbreaks characterized by high morbidity and mortality are likely associated with endothelial dysfunction. Reduction in claudin-5 or other adhesion molecules could lead to increased passage of fluid and cells into the alveolar space and result in poor gas exchange [4–6, 8]. As shown here, Poly(I:C)-induced TLR3 signaling may play a causative role in disrupting endothelial barrier function. Others have implicated changes in VE-cadherin and ZO-1 in the Poly(I:C)-mediated loss of barrier function [17, 29]. The present study shows for the first time that the effects of Poly(I:C) on claudin-5 may occur earlier and be more prominent than the changes in VE-cadherin and ZO-1 observed at later stages of barrier breakdown. In addition to the disruption of endothelial junctions, vascular leakage can be mediated by the remodeling of the actin cytoskeleton with the loss of cortical actin and an increase in central actin stress fibers that lead to cellular shape change and intercellular gaps [30]. Our data show that the reduction claudin-5 expression preceded the appearance of actin stress fibers, suggesting that Poly(I:C)-mediated actin remodeling may not play a major role in the onset of barrier dysfunction but may contribute to the progressive or late loss of barrier function.

Bronchial epithelial cells have been considered the primary target of influenza based on their expression of α-2,3-linked and α-2,6-linked sialic acid receptors, permitting binding to avian and human influenza viruses [15]. In contrast, lung endothelial cells possess α-2,3-linked sialic acid receptors as the dominant phenotype, and are susceptible to HPA1 and H5N1 viruses, but not other influenza subtypes, possibly explaining the propensity of these latter viruses to cause lung damage. Following infection with these viruses, it is likely that viral dsRNA is expressed as part of the replication cycle of the virus. In addition to the effects triggered by intracellular dsRNA in virally-infected endothelial cells, extracellular dsRNA released by virus-induced cell lysis can mediate its effects in nearby or uninfected endothelial cells. A previous study reported the detection of dsRNA in the lungs of influenza-infected rabbits [31].

TLR3 signaling plays a well-established and vital protective role in many viral infections [14–16]. However, under certain settings, abnormal or hyperactivated TLR3 responses may lead to host pathology [16]. Our findings suggest that therapeutically modulating the TLR3 pathway to reduce lung damage in cases of avian influenza, and possibly other viral pulmonary infections, may be a worthy approach to explore.

## Supporting Information

S1 Fig The effect of Poly(I:C) on VE-cadherin and ZO-1 protein and gene expression. (A) HLECs were treated with medium alone or various doses of Poly(I:C). Whole cell lysates collected at 6, 12, and 24 h were analyzed for VE-cadherin and ZO-1 expression by Western Blot. VE-cadherin (B) and ZO-1 (C) expression was normalized to β-tubulin and presented relative to control at each time point. At least three experiments were performed for each dose at each time point. Data are shown as means ± SE. *, *p*<0.05 versus control. (D and E) Effect of Poly(I:C) on *CDH5* or *TJP1* gene transcription. Cells were treated with medium alone or Poly(I:C) at 1 and 10 μg/mL for 3, 6, 12, and 24 h. RNA was collected at each time point and analyzed for *CDH5* (D) or *TJP1* (E) gene transcript relative to *GAPDH* by real-time PCR. Data were collected from three experiments for each dose at each time point and presented as means ± SE. *, p<0.05 versus control.
(TIF)

S2 Fig Poly(I:C) reduces plasma membrane staining of VE-cadherin and ZO-1 and induces central actin stress fibers. (A) HLECs grown on chamber slides were stimulated with medium (NT) or 10 μg/mL Poly(I:C) for 24 h. Immunofluorescence staining was performed using antibodies against VE-cadherin (left panel, red) and ZO-1 (right panel, red). Nuclei were counterstained with Hoechst 33342 (blue). Images are representative of three separate experiments (630x total magnification). (B) Loss of membrane claudin-5, VE-cadherin, and ZO-1 was determined by quantitating the mean fluorescence intensity at the plasma membrane with 10 μg/mL Poly(I:C) for 24 h. Data is shown as means ± SE from three separate experiments. *, *p*<0.05 versus NT. (C) Central actin stress fiber formation. HLECs grown on chamber slides were treated with medium (NT) or 10 μg/mL Poly(I:C) for 6 and 24 h. F-actin was stained with fluorescently-labeled phalloidin (green) and nuclei were counterstained with Hoechst 33342 (blue). Images are representative of three separate experiments (630x total magnification).
(TIF)

S3 Fig Poly(I:C) does not accelerate claudin-5 degradation. HLECs were treated with medium alone (NT) or 1 μg/mL Poly(I:C) for 7 h prior to the addition of 5 μg/mL cycloheximide (CHX). Whole cell lysates were collected 1 and 3 h after CHX addition and analyzed for claudin-5 by Western blot. Claudin-5 expression is presented relative to control without CHX. Means ± SE for three experiments per time point are shown.
(TIF)

S4 Fig Effect of IFNβ and IL-6 on claudin-5 expression. HLECs grown to confluence were treated with IFNβ or IL-6. (A) Whole cell lysates were collected and analyzed for claudin-5 by Western blot. Blots are representative of three experiments. (B) Claudin-5 expression was normalized to tubulin and presented relative to control. Means ± SE for three separate experiments are shown. *, *p*<0.05 versus control.
(TIF)
