# Supplementary figures and images for: Poly(I:C) Induces Human Lung Endothelial Barrier Dysfunction by Disrupting Tight Junction Expression of Claudin-5
Source: PLoS One. 2016 Aug 9;11(8):e0160875. doi: 10.1371/journal.pone.0160875 (PMC4978501; doi:10.1371/journal.pone.0160875)

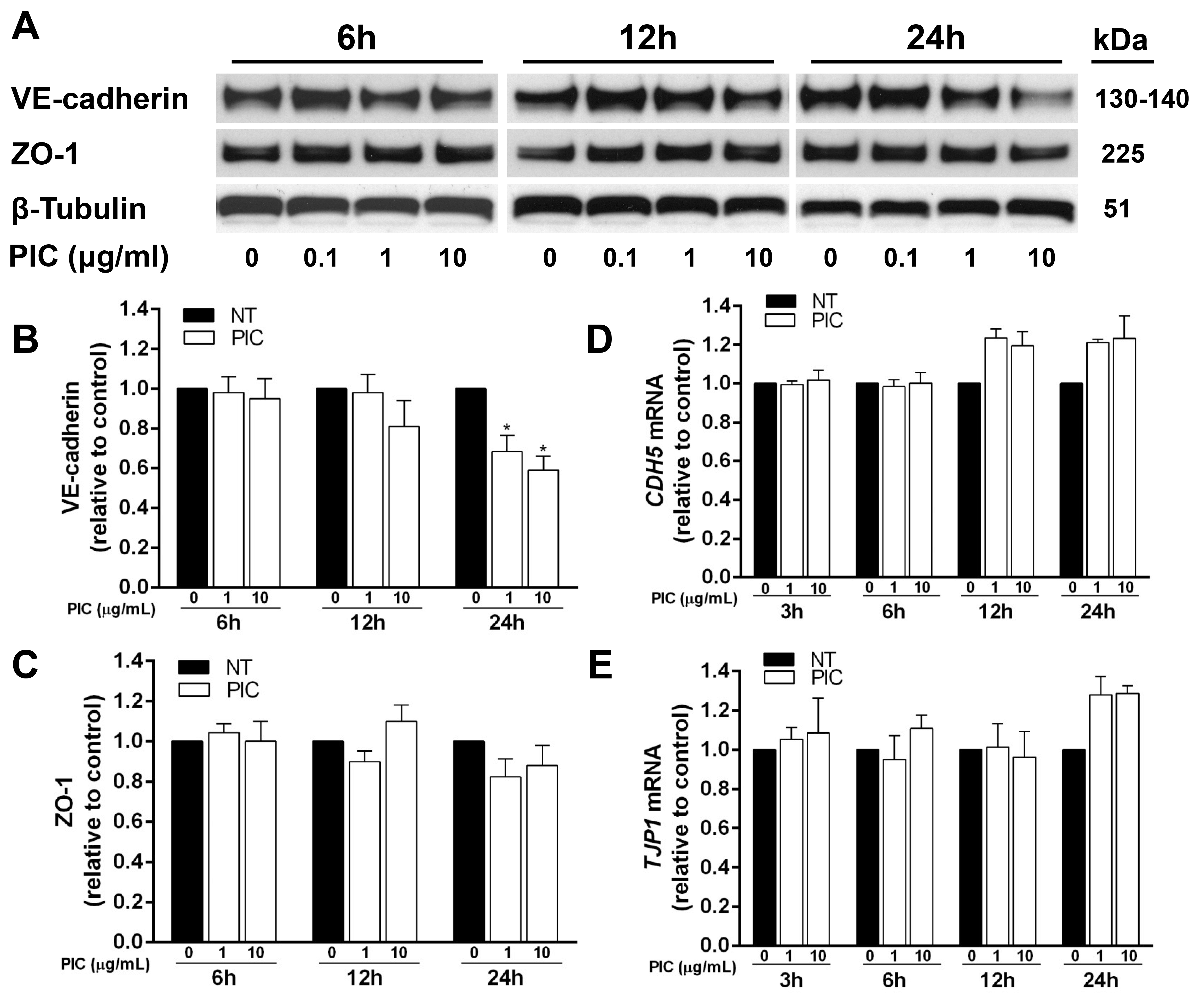

Supplement: S1 Fig — (A) HLECs were treated with medium alone or various doses of Poly(I:C). Whole cell lysates collected at 6, 12, and 24 h were analyzed for VE-cadherin and ZO-1 expression by Western Blot. VE-cadherin (B) and ZO-1 (C) expression was normalized to β-tubulin and presented relative to control at each time point. At least three experiments were performed for each dose at each time point. Data are shown as means ± SE. *, p<0.05 versus control. (D and E) Effect of Poly(I:C) on CDH5 or TJP1 gene transcription. Cells were treated with medium alone or Poly(I:C) at 1 and 10 μg/mL for 3, 6, 12, and 24 h. RNA was collected at each time point and analyzed for CDH5 (D) or TJP1 (E) gene transcript relative to GAPDH by real-time PCR. Data were collected from three experiments for each dose at each time point and presented as means ± SE. *, p<0.05 versus control. (TIF) [file pone.0160875.s001.tif]

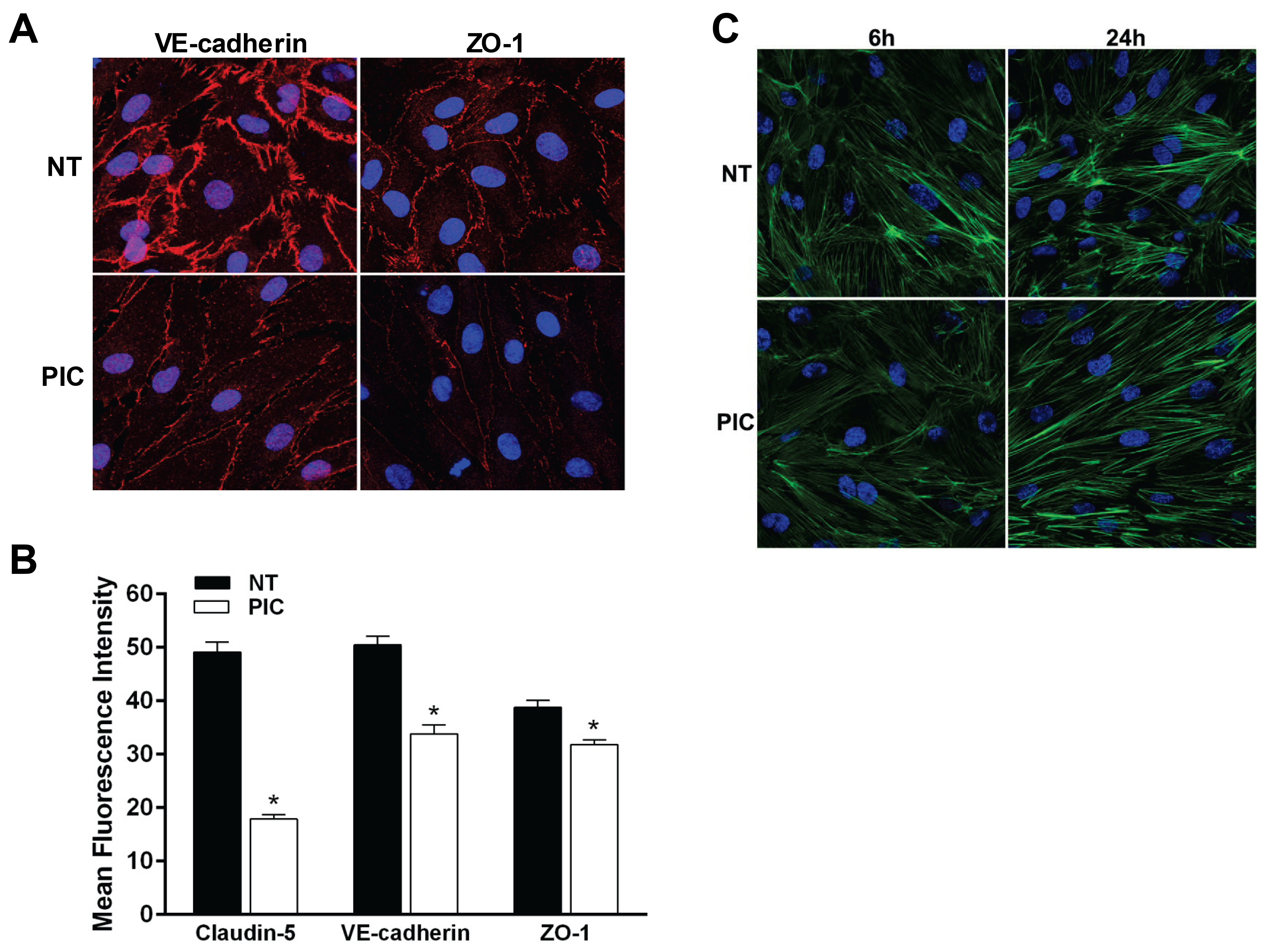

Supplement: S2 Fig — (A) HLECs grown on chamber slides were stimulated with medium (NT) or 10 μg/mL Poly(I:C) for 24 h. Immunofluorescence staining was performed using antibodies against VE-cadherin (left panel, red) and ZO-1 (right panel, red). Nuclei were counterstained with Hoechst 33342 (blue). Images are representative of three separate experiments (630x total magnification). (B) Loss of membrane claudin-5, VE-cadherin, and ZO-1 was determined by quantitating the mean fluorescence intensity at the plasma membrane with 10 μg/mL Poly(I:C) for 24 h. Data is shown as means ± SE from three separate experiments. *, p<0.05 versus NT. (C) Central actin stress fiber formation. HLECs grown on chamber slides were treated with medium (NT) or 10 μg/mL Poly(I:C) for 6 and 24 h. F-actin was stained with fluorescently-labeled phalloidin (green) and nuclei were counterstained with Hoechst 33342 (blue). Images are representative of three separate experiments (630x total magnification). (TIF) [file pone.0160875.s002.tif]

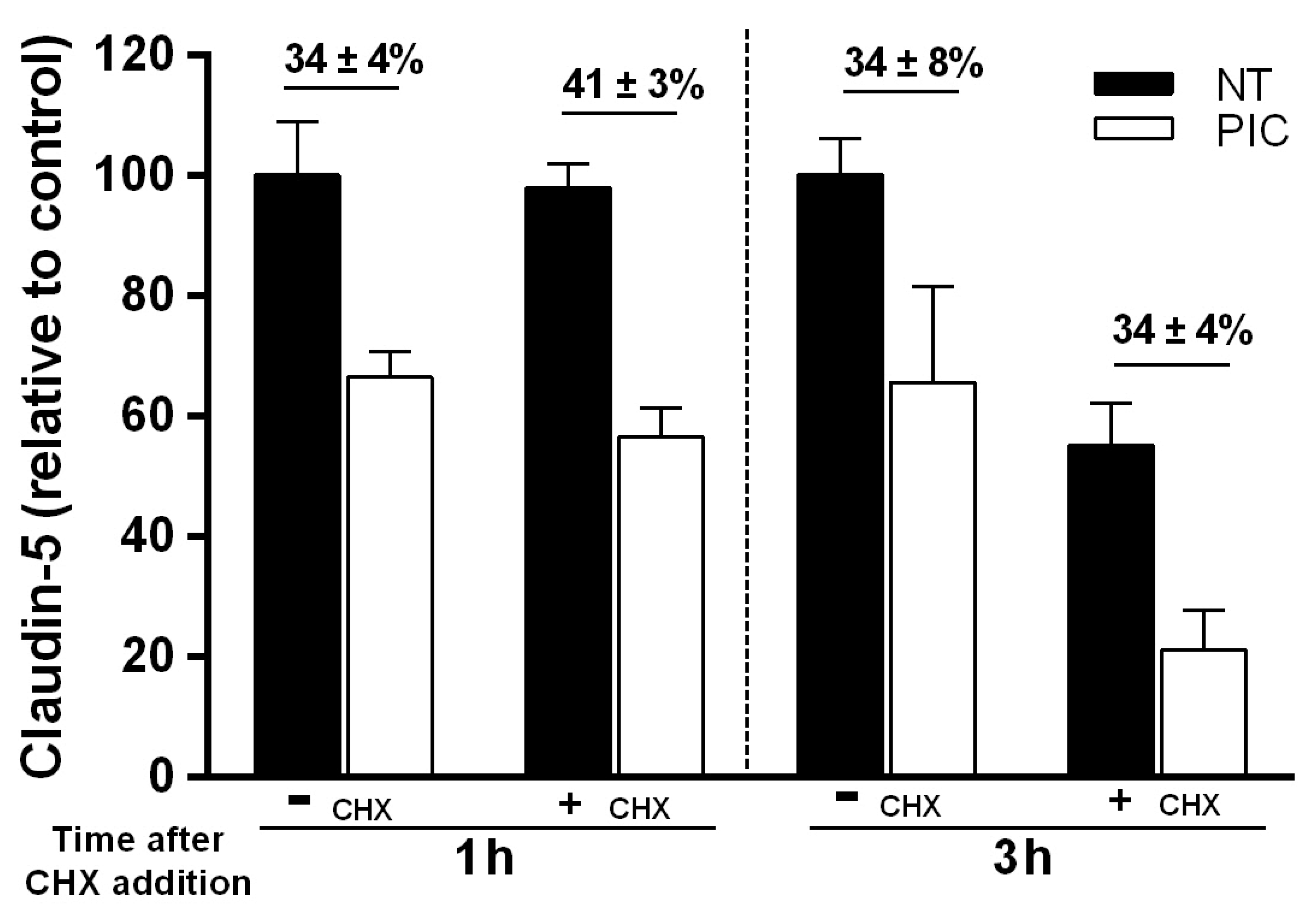

Supplement: S3 Fig — HLECs were treated with medium alone (NT) or 1 μg/mL Poly(I:C) for 7 h prior to the addition of 5 μg/mL cycloheximide (CHX). Whole cell lysates were collected 1 and 3 h after CHX addition and analyzed for claudin-5 by Western blot. Claudin-5 expression is presented relative to control without CHX. Means ± SE for three experiments per time point are shown. (TIF) [file pone.0160875.s003.tif]

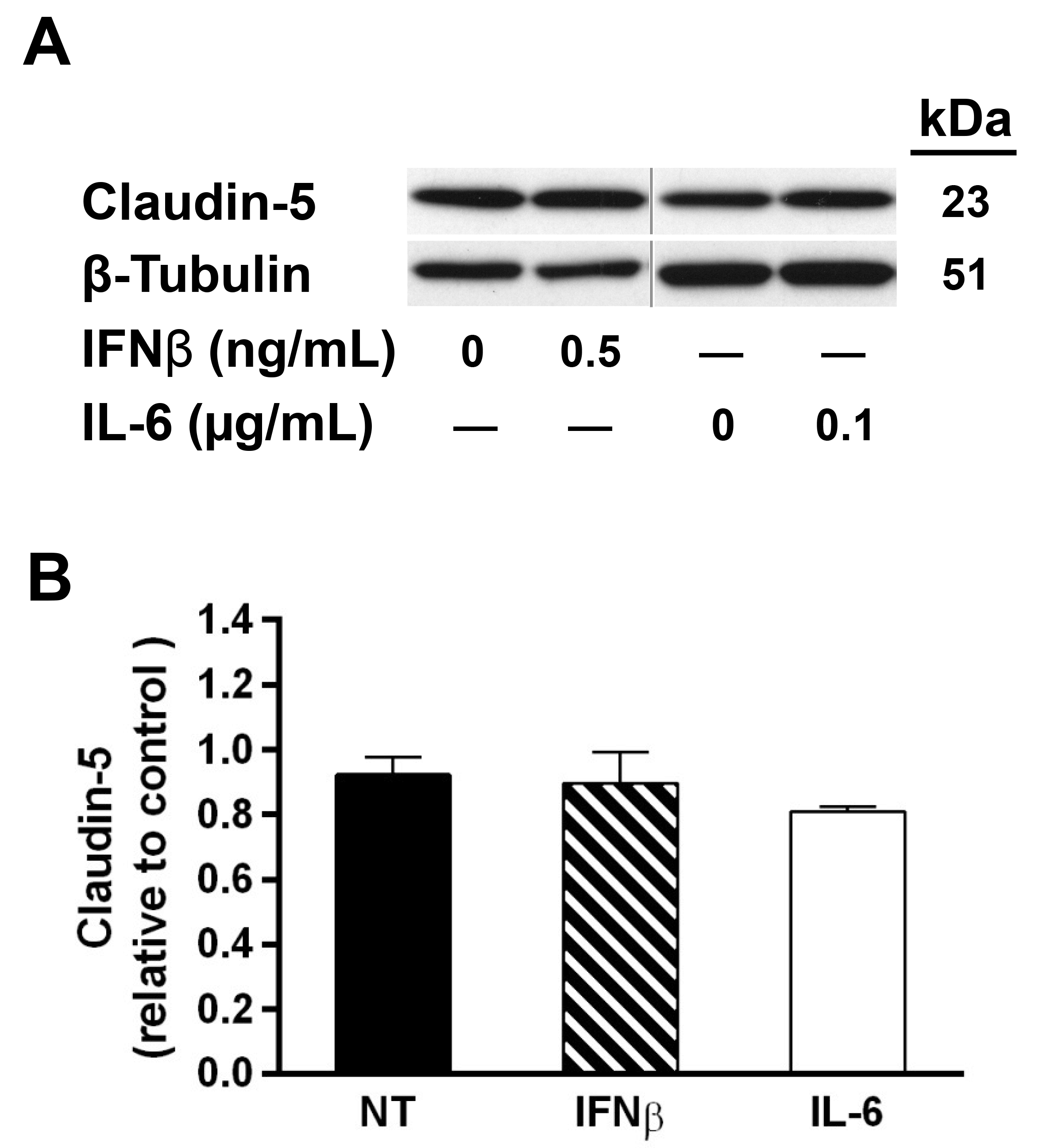

Supplement: S4 Fig — HLECs grown to confluence were treated with IFNβ or IL-6. (A) Whole cell lysates were collected and analyzed for claudin-5 by Western blot. Blots are representative of three experiments. (B) Claudin-5 expression was normalized to tubulin and presented relative to control. Means ± SE for three separate experiments are shown. *, p<0.05 versus control. (TIF) [file pone.0160875.s004.tif]
